# Synthesis, Molecular Docking, and Preclinical Evaluation of a New Succinimide Derivative for Cardioprotective, Hepatoprotective and Lipid-Lowering Effects

**DOI:** 10.3390/molecules27196199

**Published:** 2022-09-21

**Authors:** Muhammad Imran Qayyum, Sami Ullah, Umer Rashid, Abdul Sadiq, Obaidullah, Mater H. Mahnashi, Osama M. Alshehri, Mohammed M. Jalal, Khalid J. Alzahrani, Ibrahim F. Halawani

**Affiliations:** 1 Department of Pharmacy, University of Peshawar, Peshawar 25120, Pakistan; 2 Department of Pharmacy, Faculty of Medical and Health Sciences, The University of Poonch, Rawalakot, Azad Jammu & Kashmir 12350, Pakistan; 3 Department of Chemistry, Abbottabad Campus, COMSATS University Islamabad, Abbottabad 22060, Pakistan; 4 Department of Pharmacy, University of Malakand, Dir (L), Chakdara 18000, Pakistan; 5 Department of Agricultural Chemistry and Biochemistry, The University of Agriculture, Peshawar 25120, Pakistan; 6 Department of Pharmaceutical Chemistry, College of Pharmacy, Najran University, Najran 55461, Saudi Arabia; 7 Department of Clinical Laboratory Sciences, College of Applied Medical Sciences, Najran University, Najran 55461, Saudi Arabia; 8 Department of Medical Laboratory Technology, Faculty of Applied Medical Sciences, University of Tabuk, Tabuk 71491, Saudi Arabia; 9 Department of Clinical Laboratory Sciences, College of Applied Medical Sciences, Taif University, Taif 21944, Saudi Arabia

**Keywords:** cardioprotective, hepatoprotective, atenolol, succinimide, 5-FU-induced toxicity

## Abstract

Cardiac and hepatotoxicities are major concerns in the development of new drugs. Better alternatives to other treatments are being sought to protect these vital organs from the toxicities of these pharmaceuticals. In this regard, a preclinical study is designed to investigate the histopathological effects of a new succinimide derivative (Comp-1) on myocardial and liver tissues, and the biochemical effects on selected cardiac biomarkers, hepatic enzymes, and lipid profiles. For this, an initially lethal/toxic dose was determined, followed by a grouping of selected albino rats into five groups (each group had n = 6). The control group received daily oral saline for 8 days. The 5-FU (5-Fluorouracil) group received oral saline daily for 8 days, added with the administration of a single dose of 5-FU (150 mg/kg I.P.) on day 5 of the study. The atenolol group received oral atenolol (20 mg/kg) for 8 days and 5-FU (150 mg/kg I.P.) on day 5 of the protocol. Similarly, two groups of rats treated with test compound (Comp-1) were administered with 5 mg/kg I.P. and 10 mg/kg I.P. for 8 days, followed by 5-FU (150 mg/kg I.P.) on day 5. Toxicity induced by 5-FU was manifested by increases in the serum creatinine kinase myocardial band (CK-MB), troponin I (cTnI) and lactate dehydrogenase (LDH), lipid profile, and selected liver enzymes, including ALP (alkaline phosphatase), ALT (alanine transaminase), AST (aspartate aminotransferase), BT (bilirubin total), and BD (direct bilirubin). These biomarkers were highly significantly decreased after the administration of the mentioned doses of the test compound (5 mg/kg and 10 mg/kg). Similarly, histological examination revealed cardiac and hepatic tissue toxicity by 5-FU. However, those toxic effects were also significantly recovered/improved after the administration of Comp-1 at the said doses. This derivative showed dose-dependent effects and was most effective at a dose of 10 mg/kg body weight. Binding energy data computed via docking simulations revealed that our compound interacts toward the human beta2-adrenergic G protein-coupled receptor (S = −7.89 kcal/mol) with a slight stronger affinity than the calcium channel T-type (S = −7.07 kcal/mol). In conclusion, the histological and biochemical results showed that the test compound (Comp-1) had prominent cardioprotective, hepatoprotective, and lipolytic effects against 5-FU-induced toxicity in the subjected animal model.

## 1. Introduction

In cardiovascular disease (CVD), several risk factors play a pivotal role to exacerbate the pathological condition of the disease, which includes atherosclerosis, stroke, and obesity [1]. Studies have shown that myocardial infarction and stroke contribute to more than 80 percent of CVD deaths, where one-third of these deaths occur in patients having an age less than 70 years [2]. The harmfulness of the active pharmaceutical ingredients and the excipients to the cardiovascular system is a main problem facing by the pharmaceutical industry. To minimize and eliminate such toxicities, and to enhance the safety profile of the pharmaceutical ingredients, predictive screening models are greatly needed to provide better therapeutic alternatives to humans [3]. Cardiovascular toxicity, such as arrhythmias and QT interval prolongation, remains a major challenge for modern drug discovery and is one of the leading causes of drug withdrawal [4]. Clinical and nonclinical safety remains one of the main causes of drug discontinuation [5]. This abandonment can occur during preclinical or clinical development and the postapproval phase, which sometimes accounts for approximately one-third of all drug abandonments [6].

The heart and liver interact closely and affect each other [7]. Combined cardiac and hepatic dysfunctions coexist in the setting of major cardiac and hepatic diseases due to complex cardio–hepatic interactions. These organs are physiologically connected through blood circulation, which aggravates the histopathological and biochemical conditions of heart due to the liver and vice-versa. For example, congestive hepatopathy covers the spectrum of liver diseases associated with right-sided heart failure. Furthermore, ischemic reperfusion injury causes hypoperfusion, hypoxia, and reduces the oxygen supply to the liver, which may lead to cell damage. Physiologically, 20% of the cardiac output is delivered to the liver by the hepatic arterial system and portal vein, but if the portal flow decreases, adenosine is released and the hepatic artery dilates. If hypotension persists, the visceral blood flow is critically reduced and causes severe hypoxia and necrosis, which can lead to elevated bilirubin levels and alkaline phosphatase (ALP) changes [8]. Liver disorders are increasing year-by-year, with drugs being the main cause. Drug-induced liver injury depends on several factors, such as age, sex, lifestyle, obesity, nutritional status, genetic background, and dose and drug exposure [9]. Hepatotoxicity is a serious side effect associated with the development of new drugs. They are known to be among the most important toxic reactions caused by certain drugs [10]. These reactions cause poor circulation and the reduced elimination of toxic substances from the blood. It can lead to impaired immune responses to fight against infection. These toxicities may include cholestasis, deficiencies of proteins such as albumin and fibrinogen, and other metabolic disturbances in the body [11,12]. In few cases, long-term drug use can lead to damage to the cardiovascular and hepatic systems. Identifying these effects as early as possible is important for drug development [13].

Hyperlipidemia and obesity are also growing problems, partly due to the adoption of sedentary lifestyle, which includes a high intake of carbohydrates and fats together with reduced energy expenditure [14,15]. Previous evidence suggests that obesity may be associated with an increased risk of liver and heart disease. Thus, global obesity may be a major cause of disease and death in a large number of people [16,17]. It plays an important role in cardiovascular disease and in the development of atherosclerosis.

Cancer chemotherapy is associated with a significant risk of cardiac and hepatic damage during treatment [18,19]. Many anticancer therapies are associated with new-onset cardiomyopathy. Belonging to the antimetabolite class of drugs, 5-fluorouracil (as anticancer) was first introduced as a rational synthetic anticancer agent as pyrimidine analogs [20,21]. It is widely used in the treatment of many common malignancies, such as colorectal, breast, and skin cancers [22]. It produces the competitive inhibition of metabolites that have a similar structure [23]. Cardiovascular toxicity can also be observed, along with other side effects of this drug, that is manifested by effects on cardiovascular tissues and cardiac muscle [24]. Damage to the myocardial antioxidant defense system leads to the development of oxidative stress through adducts formation of the biomolecules present in the cardiac tissues [25]. Cytotoxic effects also result from the incorporation of FdUTP into DNA, as well as F-UTP and 5-fluorocytosine into RNA [26]. These metabolites are thought to affect calcium channel-dependent membrane function, interfere with mitochondrial phosphate metabolism, alter contractile proteins, cause oxidative damage and the release of vasoactive substances such as histamine and catecholamines, and trigger autoimmune mechanisms [22,23,24]. Life-threatening cardiotoxicity includes arrhythmias, ventricular tachycardia, and cardiac arrest, secondary to transmural ischemia. Risk factors associated with cardiotoxicity have received attention, providing new methods for the accurate management of chemotherapy-induced heart failure (HF). The 5-fluorouracil usually causes intrinsic hepatotoxicity through its metabolism via the microsomal enzymes and the formation of a toxic intermediate. Furthermore, thymidine phosphorylase is presumed to be involved in the induction of high triglycerides. Lipid peroxidation is also a proposed mechanism for increasing triglycerides and low-density lipids, which may also aggravate liver diseases [27].

Drug development focuses on more effective and less toxic alternatives or on reducing the toxicity of other therapeutic agents, as well as on cost-effective and safe therapies. Succinimides are organic compounds that were first introduced in 1951 for the treatment of small epilepsy. The first drug was fensuximide, followed by methsuximide, and ethosuximide [28]. The substituted succinimides contain an imide ring, and their general structure is -CO-N(R)-CO- [29]. A characteristic feature of succinimide derivatives is the addition of a new functional group to the succinimide ring, which further alters the spectroscopic and pharmacological properties of the resulting derivatives. Succinimide is part of many active molecules that have activities, such as CNS depressant [30], analgesic [31], antitumor [32], cytostatic [33], anorectic [34], nerve conduction blocking [35], antispasmodic [36], muscle relaxant [37], hypotensive [38], antibacterial [39], antifungal [40], anticonvulsant [41], and antitubercular [42] activities. The main site of action of succinimides is calcium channels in general and T-type channels in particular, which play an important role in the regulation of blood pressure and cardiac function [43,44].

The Michael addition provides a very constructive way to synthesize a variety of compounds having different structural features [45,46,47]. In the Michael additions, the donor and acceptor molecules lead to a Michael adduct [48]. A variety of Michael donors and acceptors can be used, which eventually provide a diverse array of new chiral compounds [48,49]. The chiral importance has a vital importance in drug discovery [50]. The Michael additions to the maleimide acceptors produce a succinimide product in a convenient way [51]. Using this approach, we have previously synthesized various derivatives of succinimides [48,52]. Some of our succinimide products have proved to have excellent pharmacological activities, such as analgesia, anti-inflammatory, antidiabetic, antimicrobial, and anticancer properties [51,52,53,54,55,56]. Keeping in view the potential pharmacological profile of various succinimide, herein we have synthesized a new succinimide derivative (2-(2,5-dioxo-1-phenylpyrrolidin-3-yl)-3-(4-isopropylphenyl)-2-methylpropanal), which is expected to be cardioprotective and a modulator of hepatic enzymes and lipid metabolism.

## 2. Results

### 2.1. Design Strategy

**Scheme 1:**
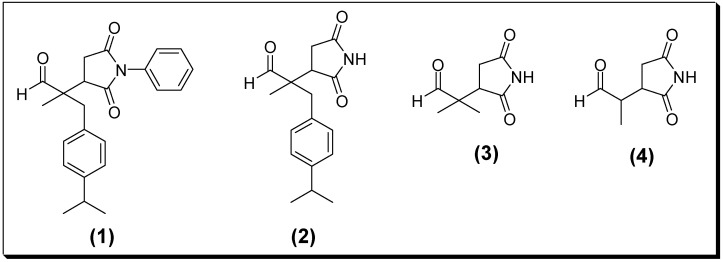
Structures of designed compounds (**1**–**4**).

As described earlier, the main site of action of succinimides are calcium channels in general and T-type channels in particular, which play an important role in the regulation of blood pressure and cardiac function. Two molecular targets, the calcium channel T-type and the human beta2-adrenergic G protein-coupled receptor, were explored. To design our target molecules, we used the 3-D structural information of the active site of the two selected molecular targets. We designed four molecules by the reaction of three different aldehydes and two N-substituted maleimides (Scheme 1). Preliminary docking studies revealed that the presence of a phenyl ring at the N-1 position and 4-isopropylphenyl)-2–methylpropanal at the 3-position are important for hydrophobic types of interaction. Moreover, the observed binding energy values for design compounds 2–4 showed that these compounds have the ability to show good experimental results. Therefore, we selected only compound 1 for further detailed experimental studies.

### 2.2. Chemistry of the Synthesized Compound

**Scheme 2:**
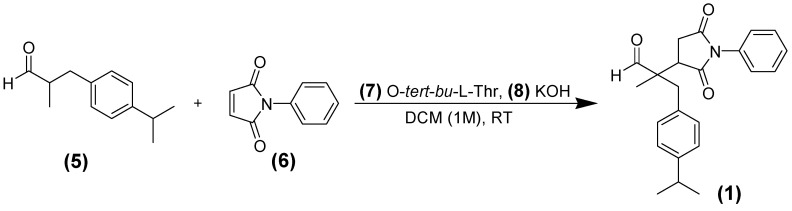
Synthesis of (2-(2,5-dioxo-1-phenylpyrrolidin-3-yl)-3-(4-isopropylphenyl)-2-methylpropanal) (Comp-1).

The synthesis of compound o) is shown in Scheme 2. Our compound (**5**) was isolated as a white solid with an R_f_ value of 0.38 in n-hexane and ethyl acetate (80:20). The isolated yield of the compound was 97%. The ^1^H and ^13^C NMR spectra are shown in (Supplementary Materials) Figures S3 and S4, respectively. The chemical purity and stereoselectivity of the compound **5** was determined with chiral HPLC analysis, as shown in S-5. The compound 5 has two different chiral centers which make a total of four stereoisomers. The four stereoisomers appeared at retention times of 23.5, 30.4, 37.2, and 43.2 min. The first two peaks (i.e., 23.5 and 30.4 min) represent the major diastereomer (*syn*), while the last two peaks (i.e., 37.2 and 43.2 min) represent the minor diastereomer (*anti*). The HPLC analysis confirmed a 3:1 diastereoselectivity and a 98% enantiomeric access.

### 2.3. Acute Toxicity

The newly synthesized compound (Comp-1) did not cause sudden death in the animals of group I (administered with a dose of 50 mg/kg) and group II (dose of 150 mg/kg). However, the animals (Balb-c mice) provided with the dose of 150 mg/kg of the test compound died within 24 h of its administration. Furthermore, the test compound also caused sudden death in group III animals (dose of 250 mg/kg). Therefore, lethal doses were expected to be more than 50 mg/kg body weight of the subjected animals.

### 2.4. Cardiac Markers

A significant increase in the level of creatinine kinase myocardial band (CK-MB), troponin I (cTnI), and lactate dehydrogenase (LDH) was observed in rats injected with 5-fluorouracil (150 mg/kg BW I.P) alone compared to the normal control. Pretreatment with atenolol (20 mg/kg BW P.O) significantly reduced the activities of these enzymes in the serum of rats receiving 5-FU. The groups treated with the test compound also significantly reduced the level of the cardiac enzymes, as shown in Table 1.

### 2.5. Liver Enzymes Analysis

A significant increase in the level of ALP (alkaline phosphatase), ALT (alanine transaminase), AST (aspartate aminotransferase), BT (bilirubin total), and BD (direct bilirubin) was observed in rats injected with 5-fluorouracil (150 mg/kg BW i.p) compared with the control group (normal saline). The results obtained for the Comp-1 test compound-treated groups highly significantly reduced the level of ALP, AST, and BT, but showed no significant effect on the liver enzymes ALT and DB at both doses (5 and 10 mg/kg). In addition, the higher dose (10 mg/kg) of the compound gave more promising results compared to 5 mg/kg, proving the dose-dependent effects of the test compound to reduce the above-mentioned liver enzymes, as shown in Table 2.

## 3. Lipid Profile

Biochemical analysis showed a significant increase in the level of total cholesterol (TC), triglycerides (TG), low-density lipids (LDL-c), very-low-density lipids (VLDL-c), and a reduction in the level of high-density lipids (HDL-c) in the animal group treated with 5-fluorouracil (150 mg/kg BW) alone compared to the normal control. The results obtained for the groups treated with the test Comp-1 showed a highly significant reduction in the levels of TC, TG, LDL-c, and VLDL-c and an increased the level of HDL-c at the dose of 10 mg/kg. However, an insignificant effect was seen regarding TG and HDL-c at the dose of 5 mg/kg. The results revealed the lipid-lowering effect (except for HDL-c) of the test compound, which was dose-dependent compared to the toxic control group, as shown in Table 3.

## 4. Histopathological Study

### 4.1. Effect on Cardiac Tissues

**Figure 1:**
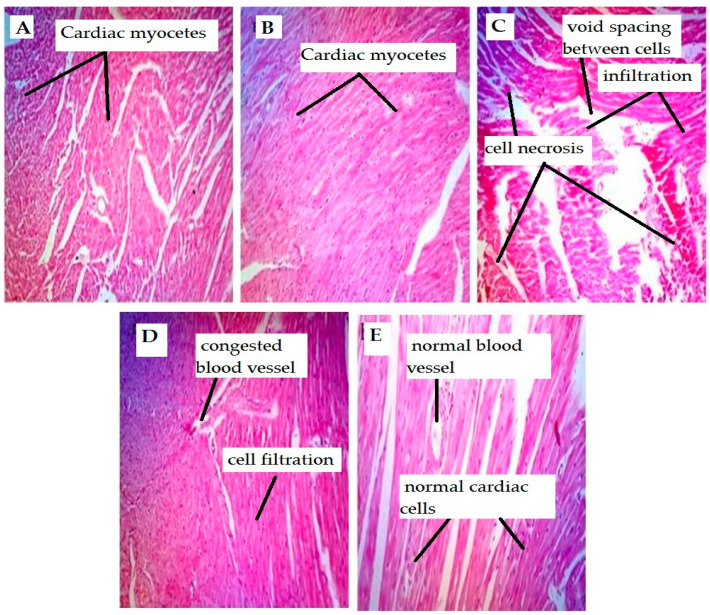
Histopathological changes induced by 5-fluorouracill and protective effect of test compounds in rat’s heart. (**A**) Cardiac section of rats belongs to control group (normal saline) showing normal morphology. (**B**) Cardiac sections from 5-fluorouracill + atenolol-treated rats revealed normal morphology with mild cellular infiltration. (**C**) Depicted a cardiac section of rats treated with 5-fluorouracill alone showing a damaged area with staining and cellular infiltration. (**D**) 5-Fluorouracill+ Comp-1 (5 mg/kg I.P) showed a reduction in degenerations. (**E**) 5-Fluorouracill + Comp-1 (10 mg/kg I.P)-treated group results in significant protection against cardiac injury caused by 5-fluorouracill. (Fixed in a 10% buffered neutral formalin solution stained with hematoxylin and eosin (H&E), magnifications power 10×.)

Histopathological evaluation (Table 4, Figure 1) of the heart tissues of the rats treated with 5-FU revealed mild-to-moderate focal and multifocal damage. Minimal to mild inflammation was observed in the animals, with myofibrillar damage and necrosis. These changes were not observed in the animals of the control group. Evaluation of cardiac tissue in the Comp-1-treated animals showed moderate focal damage and cellular infiltration at the dose of 5 mg/kg, while mild effects were seen in the animals administered with the dose of 10 mg/kg.

### 4.2. Liver Tissues

**Figure 2:**
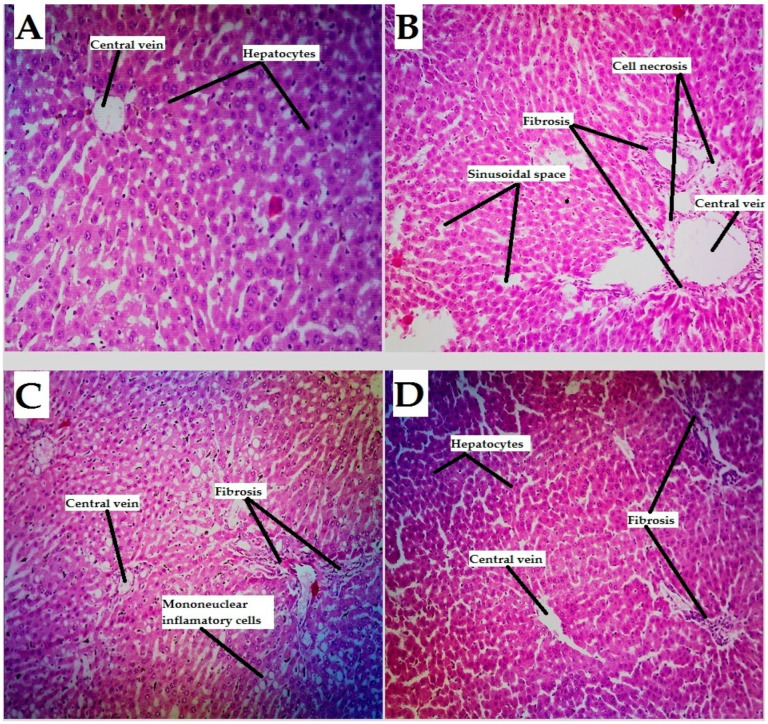
Histological section of rat’s liver. (**A**) The normal group showing the regular histological structure of the hepatic lobe. Note the normal central vein (CV) and normal hepatocytes. (**B**) 5-Fluorouracill-treated group showing centrilobular hepatocellular necrosis associated with hemorrhage and mononuclear inflammatory cell infiltration and fibrosis. (**C**) Group treated with 5-fluorouracill + Comp-1 (5 mg/kg I.P) showing hepatocyte degeneration, mononuclear inflammatory cell infiltration, and fibrosis with normal central vein. (**D**) Group treated with the formula 5-fluorouracill + Comp-1 (10 mg/kg I.P) showing hepatocyte regeneration and mild fibrosis. (Fixed in a 10% buffered neutral formalin solution Stained with hematoxylin and eosin (H&E), magnification power 10×.)

Histopathological evaluation of liver tissues of rats (Table 5, Figure 2) showed that the control group had normal lobular architecture and normal liver cells with intact cytoplasm and well-defined sinusoids. The liver section associated with 5-FU-induced toxicity showed a moderate degree of sinusoidal congestion, centrilobular necrosis with polymorphous nuclear cell infiltration, and marked vacuolations in the hepatocytes. The groups treated with Comp-1 (at doses of 5 and 10 mg/kg) against the effect of 5-FU-induced liver damage revealed protective effects resulting in the normalization of hepatocyte architecture, the absence of hepatic vacuolations, and congestion. These effects were dose-dependent, where the dose of 10 mg/kg of the test compound showed better hepatoprotective effects.

### 4.3. Docking Studies

**Figure 3:**
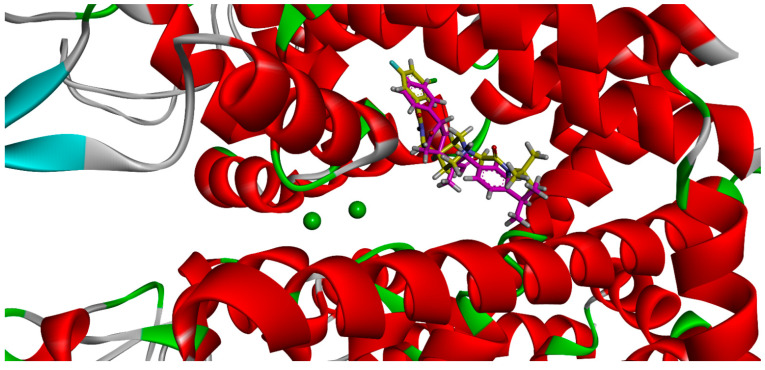
Superposed diagram of native ligand (yellow) and succinimide derivative (pink) R,S-1 in the binding site of 6KZP.

**Figure 4:**
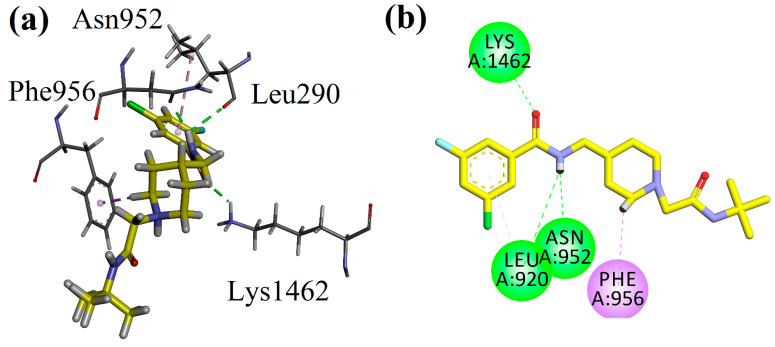
The 3-D/2/D interaction plots of native ligand in the binding site of 6KZP.

**Figure 5:**
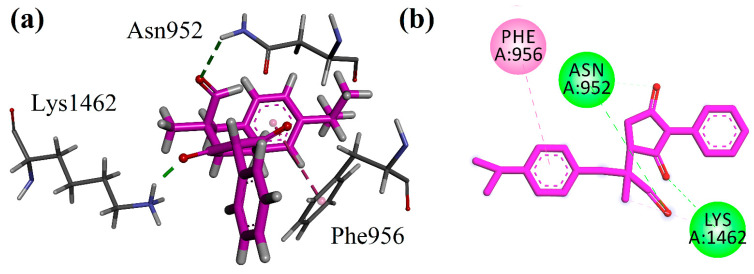
The 3-D/2/D interaction plot of R,S-1 in the binding site of 6KZP.

We analyzed the binding orientation pattern and binding energy values by using docking studies. All the possible isomers of the compounds were docked into the binding sites of the selected targets. We carried out docking simulations targeting T-type calcium channels (Ca_v_ 3.1), with the cryo-EM structure of the antagonist-bound human Ca_v_ 3.1 having the Protein Data Bank accession code 6KZP. Before the docking of the synthesized compound, we validated the docking protocol by using the redock method. Native cocrystallized ligand was extracted and then redocked in the binding site of 6KZP. The binding orientation of the redocked and experimental ligand was analyzed. The protocol showing root mean standard deviation within the limit was used for the docking simulations. The three-dimensional (3-D) superposed diagram of the native ligand and the succinimide derivative (R,S-1) are shown in Figure 3. The 3-D/2/D interaction plot of the native ligand is presented in Figure 4, which shows that it forms hydrogen bond interactions with Lys1462, Leu920, and Asn952. Phe956 forms a π-σ type of interaction, while the succinimide derivative (Comp-1) establishes hydrogen bond interactions with Asn952 and Lys1462. Phe956 also forms π-π stacking interactions (Figure 5). The computed binding energy values of the native compound of the R,S-1 in the binding site of 6KZP are −7.98 kcal/mol and −7.07 kcal/mol, respectively. The 2-D interaction plots of other isomers are presented in the Supplementary Materials (Figure S1).

**Figure 6:**
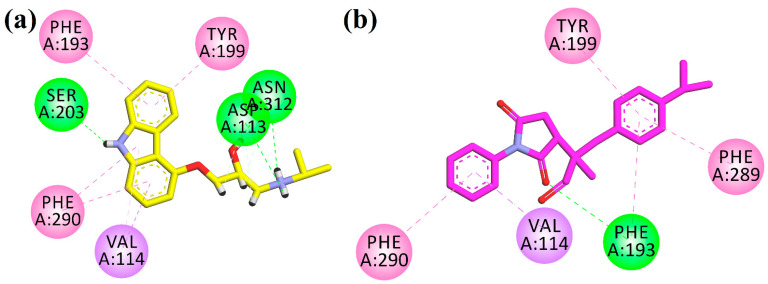
The 2D interaction plot of (**a**) native compound (yellow stick model) and (**b**) Compound R,S-1 (pink stick model) in the binding site of human beta2-adrenergic G protein-coupled receptor.

Furthermore, we analyzed the interactions of the succinimide derivative in the binding site of the human beta2-adrenergic G protein-coupled receptor. The 3D high resolution crystal structure with the PDB code 2RH1 was obtained. After the preparation and validation of the docking protocol, the synthesized Comp-1 was docked into the binding site of 2RH1. The 2D interaction plots of the native ligand and Comp-1 are shown in Figure 4. Native, (2S)-1-(9H-Carbazol-4-yloxy)-3-(isopropylamino)propan-2-ol (CAU) interacts with the active site residues Asp113, Ser203, and Asn312 via hydrogen bond interaction. Phe193, Tyr199, and Phe290 form π-π stacked interactions, while Val114 establishes a π-σ type of interaction (Figure 6a). Compound R,S-1 interacts only with one hydrogen bond interaction with Phe193. Phe193 along with Phe289 and Phe290 interact with phenyl rings via the π-π stacked interaction. Val114 also establishes a π-σ type of interaction (Figure 6b). The computed binding energy values of the native compound of the Comp-1 in the binding site of 2RH1 are −8.26 kcal/mol and −7.89 kcal/mol, respectively. The 2-D interaction plots of the other isomers are presented in the Supplementary Materials (Figure S2).

## 5. Discussion

This study was conducted to evaluate the preclinical cardioprotective, hepatoprotective, and lipid-lowering effects of a new succinimide derivative (Comp-1) using biochemical and histopathological techniques in the rat model. Toxicities were induced by the chemotherapeutic agent 5-FU. Cardiac markers including CK-MB, CTnI, and LDH, and liver enzymes such as AST, ALP, ALT, DB, and TB, were used to assess the effects, whereas TC, TG, HDL-c, LDL-c, and VLDL-c were determined as lipid profiles. In addition, histopathological examinations of cardiac and hepatic tissues were also performed to verify and correlate the histological and biochemical effects of the subjected test compound.

The results of the present study showed that the 5-FU-treated rats showed significant increases in serum CK-MB and cTnI activity and LDH levels compared to the control group. The mean value of CK-MB was 16.50 U/L for the control, while for the toxic control it was 35.50 U/L. These values showed significant differences due to the 5-FU treatment in the subjected animals. In the Comp-1 treated groups, the CK-MB values were 20.33 U/L and 13.33 U/L for 5 mg/kg and 10 mg/kg doses, respectively, which also revealed a significant reduction compared to the toxic group. Similarly, it was noted that the cTnI mean value for the control group was 0.0293 ng/mL compared with the toxic controlled value of 1.996 ng/mL (significant rise) due to the 5-FU-induced toxicity. In the Comp-1-treated groups, the cTnI values were significantly reduced to 1.529 ng/mL and 1.017 ng/mL at the given doses of 5 mg/kg and 10 mg/kg, respectively. The mean values of the LDH levels in the control group were 391.2 U/L compared with a significantly raised value in the toxic controlled group to 1061 U/L. On the other hand, in the Comp-1-treated groups, the LDH levels’ mean values were significantly reduced to 798 U/L and 362 U/L by the administration of 5 mg/kg and 10 mg/kg doses, respectively (Table 1). Histopathological studies of the cardiac tissue also showed the protective effect of the said test compound. The cardiac section of the rats belonging to the control group showed a normal morphology. Similarly, the cardiac tissues from the 5-fluorouracil + atenolol-treated rats revealed a normal morphology with a mild cellular infiltration. However, the cardiac cells treated with the 5-fluorouracil alone showed damaged areas with rapid staining and cellular infiltration. The 5-fluorouracil + Comp-1 5 mg/kg I.P showed a reduction in degenerations. Noticeably, the 5-fluorouracil + Comp-1 (at the dose of 10 mg/kg I.P)-treated group resulted in significant protection against 5-fluorouracil-induced cardiac injury, showing with just a mild infiltration (see Figure 1).

The myocardium contains high concentrations of diagnostic markers to be used as indicative parameters. Being impaired releases its contents into the extracellular fluid in a timely manner [57]. Examination of the serum CK-MB isoenzyme activity is an important diagnostic marker because of its abundance in the myocardial tissue and its sensitivity. Increased serum CK-MB isoenzyme activity reflects changes in plasma membrane integrity and permeability [58]. Cardiac troponin I (cTnI) is an intracellular structural protein specific to myocardial tissue. It is considered the gold standard for acute myocardial infarction and drug-induced cardiotoxicity. Its importance is due to the highly sensitive and specific biomarker of cardiac cell death [58]. The lactate dehydrogenase (LDH) test looks for signs of tissue damage in the body. Its function is to convert sugars into energy, so when oxygen consumption decreases, LDH levels increase [59,60]. Oxidative stress is considered an important factor in the development and progression of many chronic conditions, including cardiovascular disease [60]. This condition leads to the production of reactive oxygen species (ROS) and reactive nitrogen species (RNS). These species interfere and oxidize biological molecules such as DNA, proteins, and the lipids of membranes, leading to cell death [61]. Increased metabolism leads to ATP depletion, increased levels of superoxide ions, and reduced antioxidant capacity, as well as arterial vasoconstriction and altered plasma levels of substances involved in blood clotting and fibrinolysis [62]. As the metabolites of 5-FU are believed to influence calcium-channel-dependent membrane function, interfere with mitochondrial phosphate metabolism, alter contractile proteins, cause oxidative damage and release vasoactive substances such as histamine and catecholamines, and trigger autoimmune mechanisms [63,64,65], this mechanism may lead to cardiotoxicity, as excessive catecholamines cause increased contraction of the heart muscles. Oxidative stress damages cells, causing spasms in the coronary arteries and reducing the oxygen-carrying capacity of red blood cells, leading to myocardial ischemia, cardiac arrest, and sudden death [66]. The 5-FU-induced oxidative stress also interferes with endothelial function, which leads to reduced nitrogen oxide (NO) levels and increases the release of inflammatory cytokines. This may result in atherosclerosis in heart disease and neurological disorders [67,68]. The 5-FU-induced cardiotoxicity includes properties that include hemorrhagic infarcts, myocardial inflammatory response with interstitial fibrosis, arterial endothelial damage, and subsequent thrombosis [69]. Various compounds, natural or synthetic, possess antioxidant activities and have strong evidence to reduce the cardiac risk [70]. As succinimide derivatives possess calcium channel blocking activity, and have the capability to scavenge free radicals, as described in earlier studies [62,63], they also possess anti-inflammatory and analgesic activities [71]. The possible mechanism of the drug may lead to the blockage of the calcium channel, resulting in vasodilatation and reduced contractility, which in turn improves blood flow. Calcium-channel-blocking activity may also reduce the release of catecholamines, resulting in reduced phosphorylation and the maintenance of normal membrane function.

Serum analysis also showed elevated levels of hepatic enzymes in the 5-FU-treated group. A significant increase in the level of ALP (alkaline phosphatase), ALT (alanine transaminase), AST (aspartate aminotransferase), BT (bilirubin total), and BD (direct bilirubin) was observed in rats injected with 5-fluorouracil (150 mg/kg BW I.P) compared with the control group. The mean value of ALP for the control was 35.51 U/L, while it was 40.83 U/L in the animals treated with 5-FU. However, the Comp-1-treated groups at the doses of 5 mg/kg and 10 mg/kg resulted in a significant reduction, with the mean values of 25.68 U/L and 20.49 U/L, respectively. Similarly, the Comp-1-treated groups at the doses of 5 mg/kg and 10 mg/kg showed a significant reduction in the mean values for AST, from 37.56 U/L to 26.00 U/L and 26.17 U/L, respectively. Furthermore, there was a significant reduction in the mean values for BT, from 16.80 mg/dL to 13.88 mg/dL and 11.96 mg/dL, due to Comp-1 at the doses of 5 and 10 mg/kg, respectively. Noticeably, the test compound showed no significant effect on the liver enzymes ALT and DB at either dose compared to the 5-FU-treated group. In addition, the higher dose of 10 mg/kg of the test compound provided better output compared to 5 mg/kg, proving the dose-dependent effects of the said compound, as shown in Table 2. Histopathological examination also verified the hepatoprotective activity of the compound. The control group showed the normal histological structure of the hepatic lobe with the normal central vein (CV) and regular hepatocytes. However, the 5-fluorouracil-treated group showed centrilobular hepatocellular necrosis associated with hemorrhage and mononuclear inflammatory cell infiltration and fibrosis. The group treated with 5-fluorouracil + Comp-1 at the dose of 5 mg/kg I.P showed hepatocyte degeneration, mononuclear inflammatory cell infiltration, and fibrosis with a normal central vein. On the other hand, the group treated with the 5-fluorouracil added with Comp-1 at 10 mg/kg I.P showed significant protective effects via hepatocyte regeneration and mild fibrosis and normal central vein (Figure 2).

Biochemically, the elevated AST levels indicate liver damage, since ALT-catalyzed reactions can remodel the site and release glutamate and pyruvate; therefore, ALT is considered to be a more specific parameter for determining liver damage than AST. The elevated serum concentrations of these enzymes indicate a loss of the functional integrity of the hepatic membrane. Serum protein, total protein, ALP, and total bilirubin levels are also related to liver function [72].

As it is evident that the metabolism of the chemicals takes place predominantly in the liver, it signifies the importance and sensitivity of the organ to metabolic damage caused by drugs. Drug-induced liver damage is widespread, accounting for approximately half of all cases of acute liver failure and mimicking all forms of acute and chronic liver disease [73]. Most drugs or their metabolites associated with liver damage cause hepatotoxicity by interfering with cellular antioxidant systems, causing free radical formation and oxidative stress [74]. Hepatotoxicity with 5-fluorouracil is also a well-known phenomenon [75,76]. This damage may be caused by the inhibition of thymidylate synthase. In addition, 5-fluorouracil is extensively metabolized in the liver through microsomal enzymes, and the formation of toxic intermediates may cause liver damage [77]. Hepatic deformities, such as hepatic necrosis, vacuolated cytoplasm, congested hepatic nuclei, congested hepatic sinusoids, and inflammatory cell infiltration, are some features of 5-FU-induced liver injury [78]. Succinimide derivatives possess antioxidant activity and reduce oxidative stress by scavenging free radicals [53,55]. Its derivatives interact with the calcium channel and normalize the membrane faction [79].

A significant increase in serum total cholesterol (TC), triglycerides (TG), very-low-density lipids (VLDL-c), and low-density lipid (LDL-c) fractions, along with a decrease in high-density lipids (HDL-c), was observed in the 5-FU-treated group compared to the control group. These observed changes in the lipid profile are consistent with previously reported studies [48,74]. Treatment with the test compounds lowers TC, TAG, VLDL-c, and LDL-c levels and increases HDL-c levels significantly by the test compound at the dose of 10 mg/kg, while the 5 mg/kg dose had no significant effect on TG and HDL-c to recover the damage done in animals belonging to the toxic control group (Table 3).

Hyperglycemia due to chemotherapy can lead to vascular lesions and acute pancreatitis, so it is important to control this risk [80]. It has also been reported that 5-fluorouracil also affects the lipid profile [61,81]. Thymidine phosphorylase is presumed to be involved in the induction of high triglycerides [82]. Thymidine phosphorylase and thymidine kinase compete for thymidine, catalyzing synthetic and catabolic reactions involved in proliferation and angiogenesis [83]. Animal models showed that oxidative modification of LDL plays a major role in the development of atherosclerosis [84]. Recent studies support the theory of multiple LDL modifications and suggest that LDL particles undergo numerous modifications that alter their density, size, and chemical properties in blood flow and in the vessel wall, whereas oxidation is the final step in the overall cascade leading to atherosclerogenic properties [85]. The major cause of increased triglycerides is lipid peroxidation. As mentioned above, succinimides possess antioxidant activity which may lead to reduced reactive oxygen and increased high-density lipids. Further studies are needed to evaluate the effects and associated mechanisms of these succinimide derivatives to control oxidative stress and relevant pathogenicity.

To explore the mechanism of experimental study, we analyzed the binding orientation pattern and binding energy values by using docking studies. Two molecular targets, calcium channel T-type and the human beta2-adrenergic G protein-coupled receptor, were explored. The data revealed that Compound R,S-1 interacts toward the human beta2-adrenergic G protein-coupled receptor (S = −7.89 kcal/mol) with a slight stronger affinity than the calcium channel T-type (S = −7.07 kcal/mol).

## 6. Materials and Methods

### 6.1. Chemicals and Reagents

Dimethyl sulfoxide (DMSO), chloroform, formalin, atenolol, and 5-FU were purchased from Sigma Aldrich (St. Louis, MO, USA), while diagnostic kits for cardiac markers, liver enzymes, and lipid profiles were from Centronics, Germany, purchased through an authorized dealer. Surgical gloves, syringes, normal saline, and water for injection were purchased from the local market.

### 6.2. Synthesis of 2-(2,5-Dioxo-1-phenylpyrrolidin-3-yl) -3-(4-isopropylphenyl)-2-methylpropanal

The reaction was started by adding 1.5 equivalents of 3-(4-isopropylphenyl)-2-methylpropanal (**1**) to a small reaction vessel with catalytic amounts (5 mol %) of O-tert-bu-L-threonine (**3**) and potassium hydroxide (**4**) [86]. To this mixture was added 1 M of dichloromethane and with continued stirring at room temperature. The mixing of these reagents for 2–3 min produces the nucleophilic enamine of **1**. Afterwards, 1 equivalent of N-phenylmaleimide (**2**) was added to it with continued stirring. A time-to-time thin layer chromatographic monitoring was performed to check the progress of the reaction. After 5 h, the reaction was completed. The reaction was quenched by adding 10 mL of distilled water to it. The reaction mixture was transferred to a separating funnel by adding 10 mL of dichloromethane to it. The organic layer was separated from the water layer three times (10 mL each time). The organic layers were combined, and anhydrous sodium hydroxide was added to it. The organic layer was filtered, and the filtrate was washed with dichloromethane. The solvent was evaporated by using low vacuum rotary evaporator. The structure was confirmed by ^1^H and ^13^C NMR analyses [87]. The ^1^H NMR (400 MHz, chloroform-d): 9.58 (s, 1H), 7.49–7.45 (m, 2H), 7.41–7.37 (m, 1H), 7.32–7.25 (m, 2H), 7.19–7.07 (m, 4H), 3.27 (d, *J* = 13.55 Hz, 1H), 3.14 (dd, *J* = 6.10 and 9.47 Hz, 1H), 3.05–2.84 (m, 3H), 2.64 (dd, *J* = 6.10 and 18.21 Hz, 1H), 1.24 (d, *J* = 6.94 Hz, 6H), and 1.20 (s, 3H). The ^13^C NMR (100 MHz, chloroform-d): 203.97, 203.43, 177.75, 177.30, 175.08, 170.28, 148.04, 147.04, 133.78, 131.95, 130.78, 130.57, 130.26, 129.29, 129.02, 128.84, 128.34, 126.75, 126.70, 53.41, 52.66, 48.17, 42.64, 42.24, 40.00, 39.74, 36.31, 33.84, 32.61, 31.92, 29.87, 24.07, 24.04, 20.94, 17.19, 17.09, and 13.34. The chemical purity and stereoselectivity of the compound was determined with chiral HPLC analysis. The solvent system for the HPLC was n-heptane and isopropanol (4:1) with flow rate of 1 mL/min. The wavelength of HPLC observation was 210 nm [48].

### 6.3. Animal Breeding and Ethical Approval

The albino rats were bred and kept in the animal house of the Department of Pharmacy, University of Peshawar, at an appropriate temperature (22 ± 2 °C) with a 12 h light–dark cycle. The animals were given standard laboratory feed and water. The weight and age of the animals were determined for experimental studies. All experiments were carried out in accordance with the UK Animal Scientific Procedures Act, 1986, with the approval of the Ethical Research Committee of the Department of Pharmacy, University of Peshawar, referred to form no. 412/EC/F.LIFE,UOP-2021; dated 28 October 2021.

### 6.4. Experimental Procedures

#### 6.4.1. Acute Toxicity Study

To conduct an acute toxicity study, a total of 24 mice (BALB/c) were used for the said test compound, both genders were considered (to minimize possible sex differences associated with the results of the study), with an admissible weight of 20–30 gm, 8–12 weeks of age, maintained in a sequence of light and dark (12 h each), kept at an appropriate temperature (22 ± 2 °C). The animals were divided into three groups, where (*n* = 6). Acute toxicity was studied after intraperitoneal (I.P) injection of the test compound (Comp-1) in animals at a dose of 50 mg/kg (group I), a dose of 150 mg/kg (group II), and a dose of 250 mg/kg (group III) [88,89].

#### 6.4.2. Preclinical Observations and Survival

Untoward effects by the test compound in subjected animals were noted for toxicity and lethality. These observations included physical motor activity (movement), body appearance in different positions, such as sitting or standing, and behavioral interactions (interactions with cage mates and nesting). These observations were made throughout the acute experimental study on a daily basis [90].

#### 6.4.3. Cardioprotective Study

Mature male/female albino Sprague Dawley rats (120–150 g) were selected, housed in standard cages at room temperature, and fed with a uniform basal diet. The rats were randomly divided into five groups that received selected treatments. Animals in the control group received saline alone (orally) daily for 8 days; the FU-treated group received saline orally for 8 days followed by a single dose of 5-FU (injection of 150 mg/kg B.W.P) on day 5 of the said protocol. Two test preparations for compound at distinct doses (5 mg/kg/day/IP and 10 mg/kg/day IP) were administered for 8 days followed by a single dose of 5-FU (150 mg/kg B.W.I.P.) on day 5 of the regimen. Twenty-four hours after the last dose, blood samples were collected and all rats were killed by cervical dislocation. The serum, cardiac, and liver tissues obtained were used for histopathological and biochemical studies, including myocardial markers, liver enzymes, and lipid profiles [61].

#### 6.4.4. Biochemical Assay

Blood samples were collected following the cardiac puncture technique from each group on day 9; after centrifugation (2000× *g*) for 10 min, the separated serum was stored at 4 °C until analysis. The biochemical parameters evaluated were troponin I, creatinine kinase-MB (CK-MB), and lactate dehydrogenase (LDH), which were measured through Finecare TM analyzers. Serum glutamate pyruvate transaminase (SGPT, also known as ALT), serum glutamate oxaloacetate transaminase (SGOT, also known as AST), serum glutamate pyruvate transaminase (SGPT, also known as ALP), and serum oxaloacetate transaminase (SGOT, also known as AST) were measured using an analytical kit, Centronics, Wartenberg, Germany. Total cholesterol (TC), triglycerides (TG), high-density lipids (HDL-c), low-density lipids (LDL-c), and very-low-density lipids (VLDL-c) were measured using an analytical kit, Centronics, Germany. Details of the procedure(s) used to assess the above-stated biochemical indicators are given in Supplementary Materials. Data from these tests were used to correlate the nature and level of the toxic and protective effects on heart and liver tissues by 5-FU and test compound (Comp-1), respectively.

#### 6.4.5. Histological Study

The heart and liver were isolated after killing the rats by cervical dislocation and within 24 h of the last dose provided to subjected animals, to examine the level of cell damage and protection in each group. This assessment was used for comparison, with the toxic control group regarding the said effects. Heart and liver tissues from all experimental animals were immediately washed with normal saline and fixed in a 10% (*v*/*v*) buffered neutral formalin solution. After fixation, heart and liver tissues were processed and embedded in paraffin. Heart and liver tissues were sectioned and stained with hematoxylin and eosin (H&E). After staining, the slides were examined with a light microscope equipped with a camera (LABOMED LX400, iVu 3100, Auburn Court Fremont, CA, USA). The resulting images were evaluated for pathological changes. These lesions, which included necrosis, inflammatory cell aggregation, steatosis, fibrosis, interstitial edema, hemorrhage, and myocyte degeneration, were evaluated according to the subjective judgment of the pathologist and classified as none, few, mild, moderate, and severe.

#### 6.4.6. Docking Studies

Molecular Operating Environment (MOE 2016.0802) was used for carrying out the docking studies [90]. Docking studies were carried out on enzyme obtained from the Protein Data Bank (PDB), with 6KZP and 2RH1 accession codes, respectively. The ligand and downloaded targets were prepared by using our previously reported procedures [49,91,92,93]. The downloaded proteins were prepared and 3-D protonated by using the prepare module of MOE. Energy minimization was carried out by using Amber 10EHT force-field with 0.1 gradients, while native ligands and compounds 1–4 were prepared by using same force-field with 0.00001 gradients. For the docking simulations, active sites were determined with 10 Å of the native ligands. Docking parameters were validated by using the redocking of native ligands into the binding sites of their respective enzymes. Triangle Matcher with scoring functions London dG/Affinity dG were used as the docking method, while for Rescoring-2, the GBVI/WSA function was used. For the ligand total, ten conformations were allowed to generate, and the top-ranked conformations based on the docking score were selected for the protein–ligand interaction profile analysis. Ligand interaction and visualization was carried out via Discovery Studio Visualizer (v 2017 R2) and was used for the analysis of the docking results [94].

#### 6.4.7. Statistical Analysis

Graph-Pad Prism software version 5.01 (Graph-Pad Software, Inc., San Diego, CA, USA) was used to evaluate the results using one-way ANOVA. Values were expressed as mean ± standard deviation (SD) and standard error of mean (SEM). The *p*-values less than 0.05 were considered statistically significant.

## 7. Conclusions

From this preclinical study, it was concluded that the new succinimide derivative (Comp-1) possesses highly significant cardioprotective, hepatoprotective, and lipid-lowering effects against 5-fluorouracil-induced toxicity. Furthermore, the said protective effects of the test compound were more considerable at the dose of 10 mg/kg/day × 8 days, I.P, as compared to 5 mg/kg/day × 8 days, I.P. Binding energy data revealed that Comp-1 interacts toward the human beta2-adrenergic G protein-coupled receptor (S = −7.8923 kcal/mol) with a slight stronger affinity than the calcium channel T-type (S = −7.0706 kcal/mol).

## Figures and Tables

**Table 1:** Protective effects of Comp-1 on the activities of CK-MB, CTnl, and LDH in the serum of control and 5-FU-induced cardiotoxicity in rats.

Groups	CK-MB(U/L)	CTnI (ng/mL)	LDH (U/L)
Normal control	16.50 ± 0.619 ***	0.029 ± 0.001 ***	391.2 ± 9.495 ***
Standard drug control	17.83 ± 0.654 ***	0.085 ± 0.017 ***	604.2 ± 19.58 ***
Toxic control	35.50 ± 1.928 ***	1.99 ± 0.045 ***	1061 ± 22.20 ***
Comp-1 5 mg/kg (for 8 days)	20.33 ± 0.421 ***	1.529 ± 0.015 ***	798.2 ± 22.01 ***
Comp-1 10 mg/kg (for 8 days)	13.33 ± 0.614 ***	1.017 ± 0.031 ***	362.8 ± 13.53 ***

Values are means ± S.D for each group (*n* = 6). Normal control: normal saline; Standard drug control: atenolol-treated; Toxic control: 5-FU-treated. CK-MB (creatinine kinase–myocardial band), CTnl (troponin I), and LDH (lactate dehydrogenase). Analyzed by one-way ANOVA followed by Dunnett test. *** *p* < 0.0001. Symbols represent statistical significance. *** *p* < 0.001.

**Table 2:** Hepatoprotective effects of Comp-1 in 5-FU-induced toxicity in rats.

Groups	ALP(U/L)	ALT(U/L)	AST(U/L)	BT (mg/dL)	BD (mg/dL)
Normal control	35.51 ± 1.445 *	33.13 ± 1.504 ***	26.83 ± 0.872 ***	7.142 ± 0.200 ***	0.218 ± 0.015 ***
Standard drug Control	35.00 ± 0.966 *	38.68 ± 0.780 ***	25.00 ± 1.155 ***	8.012 ± 0.195 ***	0.366 ± 0.022 ***
Toxic control	40.83 ± 1.815 ***	52.57 ± 1.235 ***	37.56 ± 1.262 ***	16.80 ± 0.173 ***	1.907 ± 0.142 ***
Comp-1 5 mg/kg (for 8 days)	25.68 ± 1.278 ***	49.10 ± 1.865 ^ns^	26.00 ± 0.731 ***	13.88 ± 0.634 ***	2.047 ± 0.057 ^ns^
Comp-1 10 mg/kg (for 8 days)	20.49 ± 0.688 ***	49.83 ± 1.217 ^ns^	26.17 ± 1.167 ***	11.96 ± 0.963 ***	1.680 ± 0.097 ^ns^

Values are means ± S.D for each group (*n* = 6). ns = nonsignificant. Symbols represent statistical significance. * *p* < 0.05; *** *p* < 0.0001. Normal control: normal saline; Standard drug control: atenolol-treated; Toxic control: 5-FU-treated. ALP (alkaline phosphatase), ALT (alanine transaminase), AST (aspartate aminotransferase), BT (bilirubin total), and BD (direct bilirubin).

**Table 3:** Effects of Comp-1 on biochemical parameters of lipid profile in 5-FU-induced toxicity in rats.

Group	TC (mg/dL)	TG (mg/dL)	HDL-c (mg/dL)	LDL-c (mg/dL)	VLDL-c (mg/dL)
Normal control	27.95 ± 0.342 ***	113.7 ± 1.404 ***	63.12 ± 1.608 ***	78.07 ± 0.947 ***	24.27 ± 0.841 ***
Standard control	37.89 ± 0.960 ***	133.0 ± 6.005 ***	68.66 ± 2.989 ***	81.44 ± 0.989 ***	23.23 ± 0.812 ***
Toxic control	63.43 ± 0.945 ***	209.6 ± 4.037 ***	36.56 ± 1.626 ***	150.0 ± 1.891 ***	42.47 ± 0.662 ***
Comp-1 5 mg/kg (for 8 days)	23.21 ± 1.710 ***	193.1 ± 4.201 ^ns^	46.84 ± 0.497 ^ns^	121.1 ± 2.357 ***	36.21 ± 1.356 ***
Comp-1 10 mg/kg (for 8 days)	13.39 ± 1.325 ***	148.0 ± 3.856 ***	66.89 ± 2.429 ***	92.18 ± 1.433 ***	28.62 ± 1.363 ***

Values are means ± S.D for each group (*n* = 6). Normal control: normal saline; Standard drug control: atenolol-treated; Toxic control: 5-FU-treated. Total cholesterol (TC), triglycerides (TG), low-density lipids (LDL-c), very-low-density lipids (VLDL-c), and high-density lipids (HDL-c). Symbols represent statistical significance. *** *p* < 0.001. ns represents statistical not significant.

**Table 4:** Histopathological scoring of cardiac tissues control and Comp-1-treated groups (n = 6 rats in each group).

GROUPS	Normal Control (Normal Saline)	Standard Drug Control Atenolol + 5-FU-Treated Group	Toxic Control 5-FU-Treated Group	Comp-1 5 mg/kg Treated Group	Comp-1 10 mg/kg Treated Group
Cellular infiltration	0	1	2	1	1
Necrosis	0	0	3	1	0
Arterial congestion	0	0	2	1	0
Fibrosis	0	0	3	1	0

0—none, 1—mild, 2—moderate, 3—severe.

**Table 5:** Histopathological scoring of liver tissues in control and treated groups of rats.

Groups	Normal Control (Normal Saline)	Toxic Control 5-FU-Treated Group	Comp-1 5 mg/kg Treated Group	Comp-1 10 mg/kg Treated Group
Sinusoidal spaces	0	2	1	1
Necrosis	0	3	1	0
hepatocyte degeneration	0	2	1	0
Fibrosis	0	3	1	0

0—none, 1—mild, 2—moderate, 3—severe.

## Data Availability

Not Applicable.

## Supplementary Materials

The following supporting information can be downloaded at: https://www.mdpi.com/article/10.3390/molecules27196199/s1, Figure S1: 2-D interaction plots of (a) S,S-1 and (b) R,R-1 in the binding site of 6KZP; Figure S2: 2-D interaction plots of (a) S,S-1 and (b) R,R-1 in the binding site of 2RH1; Figure S3: 1H NMR spectrum of (2-(2,5-dioxo-1-phenylpyrrolidin-3-yl)-3-(4-isopropylphenyl)-2-methylpropanal); Figure S4: 13C NMR spectrum of (2-(2,5-dioxo-1-phenylpyrrolidin-3-yl)-3-(4-isopropylphenyl)-2-methylpropanal); Figure S5: Chiral HPLC chromatogram of (2-(2,5-dioxo-1-phenylpyrrolidin-3-yl)-3-(4-isopropylphenyl)-2-methylpropanal).

## Sample Availability

Sample of the compound-1 is available from the authors.

## References

[1] Jin Y., Yang C.-J., Xu X., Cao J.-N., Feng Q.-T., Yang J. (2015). MiR-214 regulates the pathogenesis of patients with coronary artery disease by targeting VEGF. Mol. Cell. Biochem..

[2] World Health Organization (2013). A Global Brief on Hypertension: Silent Killer, Global Public Health Crisis: World Health Day 2013.

[3] Zhu J.-J., Xu Y.-Q., He J.-H., Yu H.-P., Huang C.-J., Gao J.-M., Dong Q.-X., Xuan Y.-X., Li C.-Q. (2013). Human cardiotoxic drugs delivered by soaking and microinjection induce cardiovascular toxicity in zebrafish. J. Appl. Toxicol..

[4] McGrath P. (2012). Zebrafish: Methods for Assessing Drug Safety and Toxicity.

[5] Amici P. (2021). Intolerance of Uncertainty: From Transdiagnostic Model to Clinical Management. Psychiatr. Danub..

[6] Redfern W.S., Bialecki R., Ewart L., Hammond T.G., Kinter L., Lindgren S., Pollard C.E., Rolf M., Valentin J.-P. (2010). Impact and prevalence of safety pharmacology-related toxicities throughout the pharmaceutical life cycle. J. Pharmacol. Toxicol. Methods.

[7] An J., Shim J.H., Kim S.O., Lee D., Kim K.M., Lim Y.S., Lee H.C., Chung Y.H., Lee Y.S. (2014). Prevalence and prediction of coronary artery disease in patients with liver cirrhosis: A registry-based matched case–control study. Circulation.

[8] Xanthopoulos A., Starling R.C., Kitai T., Triposkiadis F. (2019). Heart failure and liver disease: Cardiohepatic interactions. JACC Heart Fail..

[9] Lei J., Dong Y., Hou Q., He Y., Lai Y., Liao C., Kawamura Y., Li J., Zhang B. (2022). Intestinal Microbiota Regulate Certain Meat Quality Parameters in Chicken. Front. Nutr..

[10] Zhang Y., Fang X.-M. (2021). Hepatocardiac or Cardiohepatic Interaction: From Traditional Chinese Medicine to Western Medicine. Evid.-Based Complement. Altern. Med..

[11] Møller S., Bernardi M. (2013). Interactions of the heart and the liver. Eur. Heart J..

[12] Samsky M.D., Patel C.B., DeWald T.A., Smith A.D., Felker G.M., Rogers J.G., Hernandez A.F. (2013). Cardiohepatic interactions in heart failure: An overview and clinical implications. J. Am. Coll. Cardiol..

[13] Henrion J., Descamps O., Luwaert R., Schapira M., Parfonry A., Heller F. (1994). Hypoxic hepatitis in patients with cardiac failure: Incidence in a coronary care unit and measurement of hepatic blood flow. J. Hepatol..

[14] Naschitz J.E., Yeshurun D., Shahar J. (1990). Cardiogenic Hepatorenal Syndrome. Angiology.

[15] Pandit A., Sachdeva T., Bafna P. (2012). Drug-induced hepatotoxicity: A review. J. Appl. Pharm. Sci..

[16] Xu T., Zhang Y., Chang P., Gong S., Shao L., Dong L. (2018). Mesenchymal stem cell-based therapy for radiation-induced lung injury. Stem Cell Res. Ther..

[17] Hunter P., Smaill B. (1988). The analysis of cardiac function: A continuum approach. Prog. Biophys. Mol. Biol..

[18] Schuster D., Laggner C., Langer T. (2005). Why Drugs Fail—A Study on Side Effects in New Chemical Entities. Curr. Pharm. Des..

[19] Varga Z.V., Ferdinandy P., Liaudet L., Pacher P. (2015). Drug-induced mitochondrial dysfunction and cardiotoxicity. Am. J. Physiol. -Heart Circ. Physiol..

[20] Lim S.S., Vos T., Flaxman A.D., Danaei G., Shibuya K., Adair-Rohani H., AlMazroa M.A., Amann M., Anderson H.R., Andrews K.G. (2012). A comparative risk assessment of burden of disease and injury attributable to 67 risk factors and risk factor clusters in 21 regions, 1990–2010: A systematic analysis for the Global Burden of Disease Study 2010. Lancet.

[21] Last A.R., Ference J.D., Menzel E.R. (2017). Hyperlipidemia: Drugs for Cardiovascular Risk Reduction in Adults. Am. Fam. Physician.

[22] Schmitz G., Orsó E. (2015). Lipoprotein(a) hyperlipidemia as cardiovascular risk factor: Pathophysiological aspects. Clin. Res. Cardiol. Suppl..

[23] Payne D.L., Nohria A. (2017). Prevention of Chemotherapy Induced Cardiomyopathy. Curr. Heart Fail. Rep..

[24] Swain S.M., Whaley F.S., Ewer M.S. (2003). Congestive heart failure in patients treated with doxorubicin: A retrospective analysis of three trials. Cancer Interdiscip. Int. J. Am. Cancer Soc..

[25] More L.A., Lane S., Asnani A. (2021). 5-FU Cardiotoxicity: Vasospasm, Myocarditis, and Sudden Death. Curr. Cardiol. Rep..

[26] Chen J., Zou Q., Li J. (2021). DeepM6ASeq-EL: Prediction of human N6-methyladenosine (m6A) sites with LSTM and ensemble learning. Front. Comput. Sci..

[27] Longley D.B., Harkin D.P., Johnston P.G. (2003). 5-Fluorouracil: Mechanisms of action and clinical strategies. Nat. Rev. Cancer.

[28] Teschendorf H.J., Kretzschmar R. (1985). Succinimides. Antiepileptic Drugs.

[29] Patil M.M., Rajput S.S. (2014). Succinimides: Synthesis, reaction, and biological activity. Int. J. Pharm. Pharm. Sci..

[30] Aeberli P., Gogerty J.H., Houlihan W.J., Iorio L.C. (1976). Synthesis and central nervous system depressant activity of some bicyclic amides. J. Med. Chem..

[31] Corrêa R., Filho V.C., Rosa P.W., Pereira C.I., Schlemper V., Nunes R.J. (1997). Synthesis of new succinimides and sulphonated derivatives with analgesic action in mice. Pharm. Pharmacol. Commun..

[32] Crider A.M., Kolczynski T.M., Yates K.M. (1980). Synthesis and anticancer activity of nitrosourea derivatives of phensuximide. J. Med. Chem..

[33] Hall I.H., Wong O.T., Scovill J.P. (1995). The cytotoxicity of N-pyridinyl and N-quinolinyl substituted derivatives of phthalimide and succinimide. Biomed. Pharmacother..

[34] Rich D.H., Gardner J.H. (1983). Synthesis of the cytostatic cyclic tetrapeptide, chlamydocin. Tetrahedron Lett..

[35] Kaczorowski G.J., McManus O.B., Priest B.T., Garcia M.L. (2008). Ion Channels as Drug Targets: The Next GPCRs. J. Gen. Physiol..

[36] Filho V.C., Nunes R.J., Calixto J.B., Yunes R.A. (1995). Inhibition of guinea-pig ileum contraction by phyllanthimide analogues: Structure-activity relationships. Pharm. Pharmacol. Commun..

[37] Musso D.L., Cochran F.R., Kelley J.L., McLean E.W., Selph J.L., Rigdon G.C., Orr G.F., Davis R.G., Cooper B.R., Styles V.L. (2002). Indanylidenes. 1. Design and Synthesis of (E)-2-(4,6-Difluoro-1-indanylidene)acetamide, a Potent, Centrally Acting Muscle Relaxant with Antiinflammatory and Analgesic Activity. J. Med. Chem..

[38] Coram W.M., Brezenoff H.E. (1983). The antihypertensive effect of a selective central muscarinic cholinergic antagonist: N-(4-diethylamino-2-butynyl)-succinimide. Drug Dev. Res..

[39] Zentz F., Valla A., Le Guillou R., Labia R., Mathot A.-G., Sirot D. (2002). Synthesis and antimicrobial activities of N-substituted imides. II Farm..

[40] Hazra B., Pore V., Dey S., Datta S., Darokar M., Saikia D., Khanuja S.P., Thakur A. (2004). Bile acid amides derived from chiral amino alcohols: Novel antimicrobials and antifungals. Bioorg. Med. Chem. Lett..

[41] Kornet M.J., Crider A.M., Magarian E.O. (1977). Potential long-acting anticonvulsants. 1. Synthesis and activity of succinimides containing an alkylating group at the 2 position. J. Med. Chem..

[42] Isaka M., Prathumpai W., Wongsa P., Tanticharoen M., Tanticharoen M. (2006). Hirsutellone F, a Dimer of Antitubercular Alkaloids from the Seed Fungus *Trichoderma* Species BCC 7579. ChemInform.

[43] Das N. (2021). “Aberrant” neuronal stimulation and” cannabis psychosis”-hypothesis to a biological plausibility!. Psychiatr. Danub..

[44] Tang C.-M., Presser F., Morad M. (1988). Amiloride Selectively Blocks the Low Threshold (T) Calcium Channel. Science.

[45] Ahmad S., Mahnashi M.H., Alyami B.A., Alqahtani Y.S., Ullah F., Ayaz M., Tariq M., Sadiq A., Rashid U. (2021). Synthesis of Michael Adducts as Key Building Blocks for Potential Analgesic Drugs: In vitro, in vivo and in silico Explorations. Drug Des. Dev. Ther..

[46] Nugent T.C., Bibi A., Sadiq A., Shoaib M., Umar M.N., Tehrani F.N. (2012). Chiral picolylamines for Michael and aldol reactions: Probing substrate boundaries. Org. Biomol. Chem..

[47] Sadiq A., Nugent T.C. (2020). Catalytic Access to Succinimide Products Containing Stereogenic Quaternary Carbons. ChemistrySelect.

[48] Nugent T.C., Sadiq A., Bibi A., Heine T., Zeonjuk L.L., Vankova N., Bassil B.S. (2012). Noncovalent bifunctional organocatalysts: Powerful tools for contiguous quaternary-tertiary stereogenic carbon formation, scope, and origin of enantioselectivity. Chem. Eur. J..

[49] Jan M.S., Ahmad S., Hussain F., Ahmad A., Mahmood F., Rashid U., Abid O.-U., Ullah F., Ayaz M., Sadiq A. (2020). Design, synthesis, in-vitro, in-vivo and in-silico studies of pyrrolidine-2,5-dione derivatives as multitarget anti-inflammatory agents. Eur. J. Med. Chem..

[50] Nugent T.C., Negru D.E., El-Shazly M., Hu D., Sadiq A., Bibi A., Umar M.N. (2011). Sequential Reductive Amination-Hydrogenolysis: A One-Pot Synthesis of Challenging Chiral Primary Amines. Adv. Synth. Catal..

[51] Sadiq A., Mahnashi M.H., Alyami B.A., Alqahtani Y.S., Alqarni A.O., Rashid U. (2021). Tailoring the substitution pattern of Pyrrolidine-2,5-dione for discovery of new structural template for dual COX/LOX inhibition. Bioorg. Chem..

[52] Mahnashi M.H., Alyami B.A., Alqahtani Y.S., Alqarni A.O., Jan M.S., Hussain F., Zafar R., Rashid U., Abbas M., Tariq M. (2022). Antioxidant Molecules Isolated from Edible Prostrate Knotweed: Rational Derivatization to Produce More Potent Molecules. Oxid. Med. Cell. Longev..

[53] Huneif M.A., Alshehri D.B., Alshaibari K.S., Dammaj M.Z., Mahnashi M.H., Majid S.U., Javed M.A., Ahmad S., Rashid U., Sadiq A. (2022). Design, synthesis and bioevaluation of new vanillin hybrid as multitarget inhibitor of α-glucosidase, α-amylase, PTP-1B and DPP4 for the treatment of type-II diabetes. Biomed. Pharm..

[54] Sadiq A., Mahmood F., Ullah F., Ayaz M., Ahmad S., Haq F.U., Khan G., Jan M.S. (2015). Synthesis, anticholinesterase and antioxidant potentials of ketoesters derivatives of succinimides: A possible role in the management of Alzheimer’s. Chem. Central J..

[55] Mahmood F., Jan M.S., Ahmad S., Rashid U., Ayaz M., Ullah F., Hussain F., Ahmad A., Khan A.U., Aasim M. (2017). Ethyl 3-oxo-2-(2, 5-dioxopyrrolidin-3-yl) butanoate derivatives: Anthelmintic and cytotoxic potentials, antimicrobial, and docking studies. Front. Chem.

[56] Ahmad A., Ullah F., Sadiq A., Ayaz M., Rahim H., Rashid U., Ahmad S., Jan M.S., Ullah R., Shahat A.A. (2019). Pharmacological Evaluation of Aldehydic-Pyrrolidinedione Against HCT-116, MDA-MB231, NIH/3T3, MCF-7 Cancer Cell Lines, Antioxidant and Enzyme Inhibition Studies. Drug Des. Dev. Ther..

[57] Panteghini M., Pagani F., Cuccia C. (1987). Activity of serum aspartate aminotransferase isoenzymes in patients with acute myocardial infarction. Clin. Chem..

[58] Farvin K.H.S., Anandan R., Kumar S.H.S., Shiny K.S., Sankar T.V., Thankappan T.K. (2004). Effect of squalene on tissue defense system in isoproterenol-induced myocardial infarction in rats. Pharmacol. Res..

[59] Hammond G.L., Nadal-Ginard B., Talner N.S., Markert C.L. (1976). Myocardial LDH isozyme distribution in the ischemic and hypoxic heart. Circulation.

[60] Liguori I., Russo G., Curcio F., Bulli G., Aran L., Della-Morte D., Gargiulo G., Testa G., Cacciatore F., Bonaduce D. (2018). Oxidative stress, aging, and diseases. Clin. Interv. Aging.

[61] Forman H.J., Zhang H. (2021). Targeting oxidative stress in disease: Promise and limitations of antioxidant therapy. Nat. Rev. Drug Discov..

[62] Mohamed E.T., Safwat G.M. (2016). Evaluation of cardioprotective activity of Lepidium sativum seed powder in albino rats treated with 5-fluorouracil. Beni-Suef Univ. J. Basic Appl. Sci..

[63] Cianci G., Morelli M.F., Cannita K., Morese R., Ricevuto E., Di Rocco Z.C., Porzio G., Baldi P.L., Ficorella C. (2003). Prophylactic options in patients with 5-fluorouracil-associated cardiotoxicity. Br. J. Cancer.

[64] Ficorella C., Ricevuto E., Morelli M.F., Morese R., Cannita K., Cianci G., Porzio G., Di Rocco Z.C., De Galitiis F., De Tursi M. (2006). Increased tolerability of bimonthly 12-hour timed flat infusion 5-fluorouracil/irinotecan regimen in advanced colorectal cancer: A dose-finding study. Oncol. Rep..

[65] Parker W.B., Cheng Y.C. (1990). Metabolism and mechanism of action of 5-fluorouracil. Pharmacol. Ther..

[66] Polk A., Vistisen K., Vaage-Nilsen M., Nielsen D.L. (2014). A systematic review of the pathophysiology of 5-fluorouracil-induced cardiotoxicity. BMC Pharmacol. Toxicol..

[67] Tousoulis D., Psaltopoulou T., Androulakis E., Papageorgiou N., Papaioannou S., Oikonomou E., Synetos A., Stefanadis C. (2014). Oxidative Stress and Early Atherosclerosis: Novel Antioxidant Treatment. Cardiovasc. Drugs Ther..

[68] Leitão R.F.C., Ribeiro R.A., Bellaguarda E.A.L., Macedo F.D.B., Silva L.R., Oriá R., Vale M., Cunha F.Q., Brito G.A.C. (2006). Role of nitric oxide on pathogenesis of 5-fluorouracil induced experimental oral mucositis in hamster. Cancer Chemother. Pharmacol..

[69] Hou Q., Huang J., Xiong X., Guo Y., Zhang B. (2022). Role of Nutrient-sensing Receptor GPRC6A in Regulating Colonic Group 3 Innate Lymphoid Cells and Inflamed Mucosal Healing. J. Crohn’s Colitis.

[70] Bertolini A., Fiumanò M., Fusco O., Muffatti A., Scarinci A., Pontiggia G., Scopelliti M. (2001). Acute cardiotoxicity during capecitabine treatment: A case report. Tumori J..

[71] Hamilton K.L. (2007). Antioxidants and cardioprotection. Med. Sci. Sports Exerc..

[72] Cieślak M., Napiórkowska M., Kaźmierczak-Barańska J., Królewska-Golińska K., Hawrył A., Wybrańska I., Nawrot B. (2021). New Succinimides with Potent Anticancer Activity: Synthesis, Activation of Stress Signaling Pathways and Characterization of Apoptosis in Leukemia and Cervical Cancer Cells. Int. J. Mol. Sci..

[73] Shehab N.G., Abu-Gharbieh E., Bayoumi F.A. (2015). Impact of phenolic composition on hepatoprotective and antioxidant effects of four desert medicinal plants. BMC Complement. Altern. Med..

[74] Reid A.B., Kurten R.C., McCullough S.S., Brock R.W., Hinson J.A. (2004). Mechanisms of Acetaminophen-Induced Hepatotoxicity: Role of Oxidative Stress and Mitochondrial Permeability Transition in Freshly Isolated Mouse Hepatocytes. J. Pharmacol. Exp. Ther..

[75] Kaplowitz N. (2001). Drug-induced liver disorders. Drug Saf..

[76] Zeng D., Wang Y., Chen Y., Li D., Li G., Xiao H., Hou J., Wang Z., Hu L., Wang L. (2021). Angelica Polysaccharide Antagonizes 5-FU-Induced Oxidative Stress Injury to Reduce Apoptosis in the Liver Through Nrf2 Pathway. Front. Oncol..

[77] Benincasa G., Cuomo O., Vasco M., Vennarecci G., Canonico R., Della Mura N., Alfano R., Napoli C. (2020). Epigenetic-sensitive challenges of cardiohepatic interactions: Clinical and therapeutic implications in heart failure patients. Eur. J. Gastroenterol. Hepatol..

[78] Hoofnagle J.H., Serrano J., Knoben J.E., Navarro V.J. (2012). LiverTox: A website on drug-induced liver injury. Hepatology.

[79] Abou-Zeid N.R. (2014). Ameliorative effect of vitamin C against 5-fuorouracil-induced hepatotoxicity in mice: A light and electron microscope study. J. Basic Appl. Zool..

[80] Krivoshein A.V. (2016). Antiepileptic drugs based on the α-substituted amide group pharmacophore: From chemical crystallography to molecular pharmaceutics. Curr. Pharm. Des..

[81] Saito Y., Takekuma Y., Komatsu Y., Sugawara M. (2020). Hypertriglyceridemia induced by S-1: A novel case report and review of the literature. J. Oncol. Pharm. Pract..

[82] Abdel-Hamid H.F., Soliman A., Helaly F.M., Ragab S. (2011). Cytotoxic potency and induced biochemical parameters in mice serum of new furan derivatives against liver cancer cell line. Acta Pol. Pharm. -Drug Res..

[83] Javot L., Spaëth D., Scala-Bertola J., Gambier N., Petitpain N., Gillet P. (2011). Severe hypertriglyceridaemia during treatment with capecitabine. Br. J. Cancer.

[84] Brockenbrough J.S., Morihara J.K., Hawes S.E., Stern J.E., Rasey J.S., Wiens L.W., Feng Q., Vesselle H. (2009). Thymidine Kinase 1 and Thymidine Phosphorylase Expression in Non-Small-cell Lung Carcinoma in Relation to Angiogenesis and Proliferation. J. Histochem. Cytochem..

[85] Steinberg D. (2009). The LDL modification hypothesis of atherogenesis: An update. J. Lipid Res..

[86] Mahmood F., Khan J.A., Mahnashi M.H., Jan M.S., Javed M.A., Rashid U., Sadiq A., Hassan S.S., Bungau S. (2022). Anti-Inflammatory, Analgesic and Antioxidant Potential of New (2 S, 3 S)-2-(4-isopropylbenzyl)-2-methyl-4-nitro-3-phenylbutanals and Their Corresponding Carboxylic Acids through In Vitro, In Silico and In Vivo Studies. Molecules.

[87] Sadiq A., Mahnashi M.H., Rashid U., Jan M.S., Alshahrani M.A., Huneif M.A. (2022). 3-(((1S,3S)-3-((R)-hydroxy(4-(trifluoromethyl)phenyl)methyl)-4-oxocyclohexyl)methyl)pentane-2,4-dione: Design and synthesis of new stereopure multi-target antidiabetic agent. Molecules.

[88] Poznyak A.V., Nikiforov N.G., Markin A.M., Kashirskikh D.A., Myasoedova V.A., Gerasimova E.V., Orekhov A.N. (2021). Overview of OxLDL and Its Impact on Cardiovascular Health: Focus on Atherosclerosis. Front. Pharmacol..

[89] Bhat M.A., Al-Omar M.A., Khan A.A., Alanazi A.M., Naglah A.M. (2019). Synthesis and antihepatotoxic activity of dihydropyrimidinone derivatives linked with 1,4-benzodioxane. Drug Des. Dev. Ther..

[90] Kamil M., Fatima A., Ullah S., Ali G., Khan R., Ismail N., Qayum M., Irimie M., Dinu C., Ahmedah H. (2021). Toxicological Evaluation of Novel Cyclohexenone Derivative in an Animal Model through Histopathological and Biochemical Techniques. Toxics.

[91] Malik H.N., Jabeen A., Ashraf S., Haq Z.U., Salar U., Arshia, Khan K.M. (2022). Benzophenone and coumarin derivatives as 3-CLPro inhibitors: Targeting cytokine storm through in silico and in vitro approaches. J. Mol. Struct..

[92] Tanoli S.T., Ramzan M., Hassan A., Sadiq A., Jan M.S., Khan F.A., Ullah F., Ahmad H., Bibi M., Mahmood T. (2018). Design, synthesis and bioevaluation of tricyclic fused ring system as dual binding site acetylcholinesterase inhibitors. Bioorg. Chem..

[93] Bibi M., Qureshi N.A., Sadiq A., Farooq U., Hassan A., Shaheen N., Asghar I., Umer D., Ullah A., Khan F.A. (2020). Exploring the ability of dihydropyrimidine-5-carboxamide and 5-benzyl-2,4-diaminopyrimidine-based analogues for the selective inhibition of L. major dihydrofolate reductase. Eur. J. Med. Chem..

[94] Javed M.A., Ashraf N., Saeed J.M., Mahnashi M.H., Alqahtani Y.S., Alyami B.A., Alqarni A.O., Asiri Y.I., Ikram M., Sadiq A. (2021). Structural Modification, In Vitro, In Vivo, Ex Vivo, and In Silico Exploration of Pyrimidine and Pyrrolidine Cores for Targeting Enzymes Associated with Neuroinflammation and Cholinergic Deficit in Alzheimer’s Disease. ACS Chem. Neurosci..

