# Supplementary material for: Synthesis, Molecular Docking, and Preclinical Evaluation of a New Succinimide Derivative for Cardioprotective, Hepatoprotective and Lipid-Lowering Effects
Source: Molecules. 2022 Sep 21;27(19):6199. doi: 10.3390/molecules27196199 (PMC9573045; doi:10.3390/molecules27196199)
Supplement: Supplementary file 1 [file molecules-27-06199-s001.zip › molecules-1810960-supplementary.pdf]

SUPPLEMENTARY DATA OF THE MANUSCRIPT ID: MOLECULES-1810960

SYNTHESIS, MOLECULAR DOCKING AND PRECLINICAL EVALUATION OF NEW SUCCINIMIDE DERIVATIVE FOR CARDIOPROTECTIVE, HEPATOPROTECTIVE AND LIPID LOWERING EFFECTS

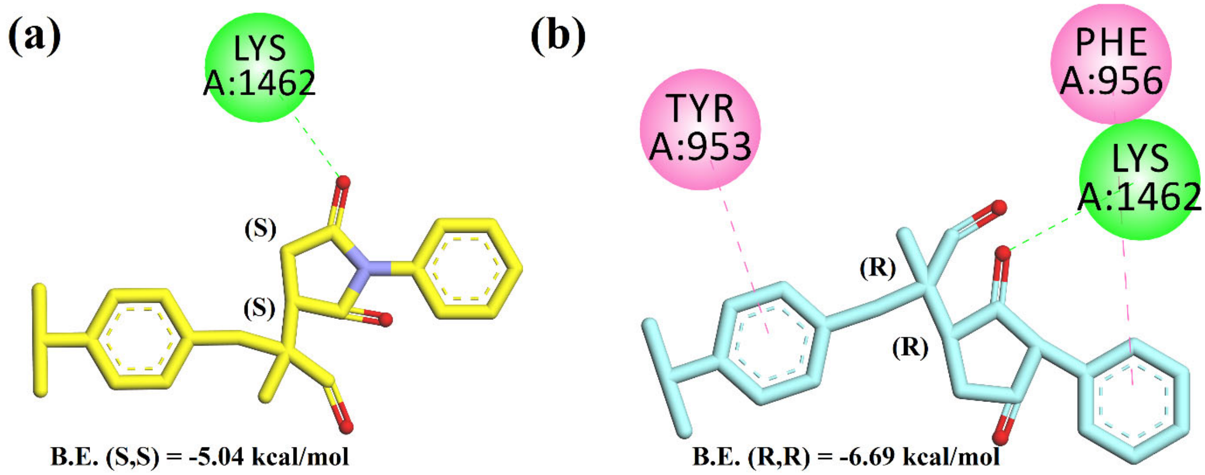

Figure S1: 2-D interaction plots of (a) S,S-1 and (b) R,R-1 in the binding site of 6KZP

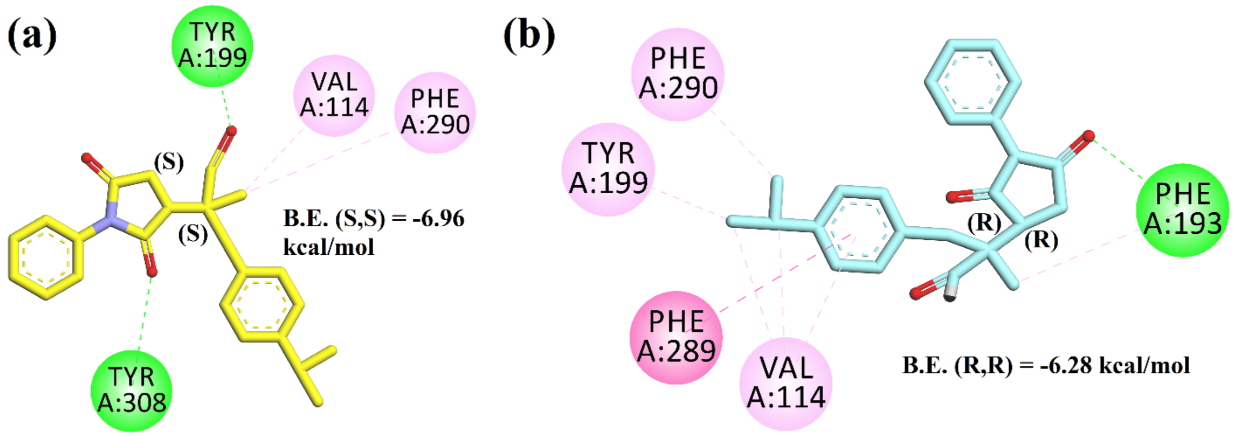

Figure S2: 2-D interaction plots of (a) S,S-1 and (b) R,R-1 in the binding site of 2RH1

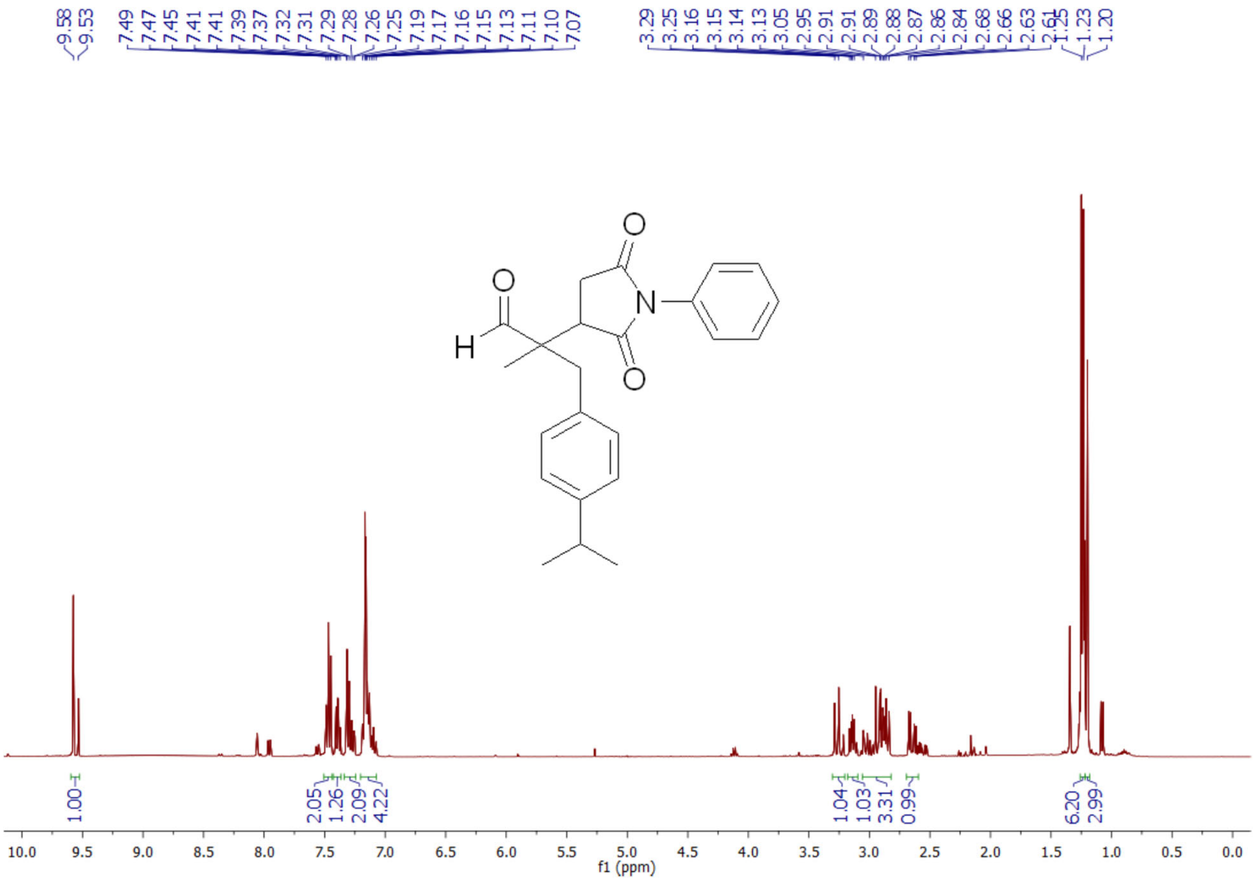

Figure S3. ^{1}H NMR spectrum of (2-(2,5-dioxo-1-phenylpyrrolidin-3-yl)-3-(4-isopropylphenyl)-2-methylpropanal) (**compd-1**).

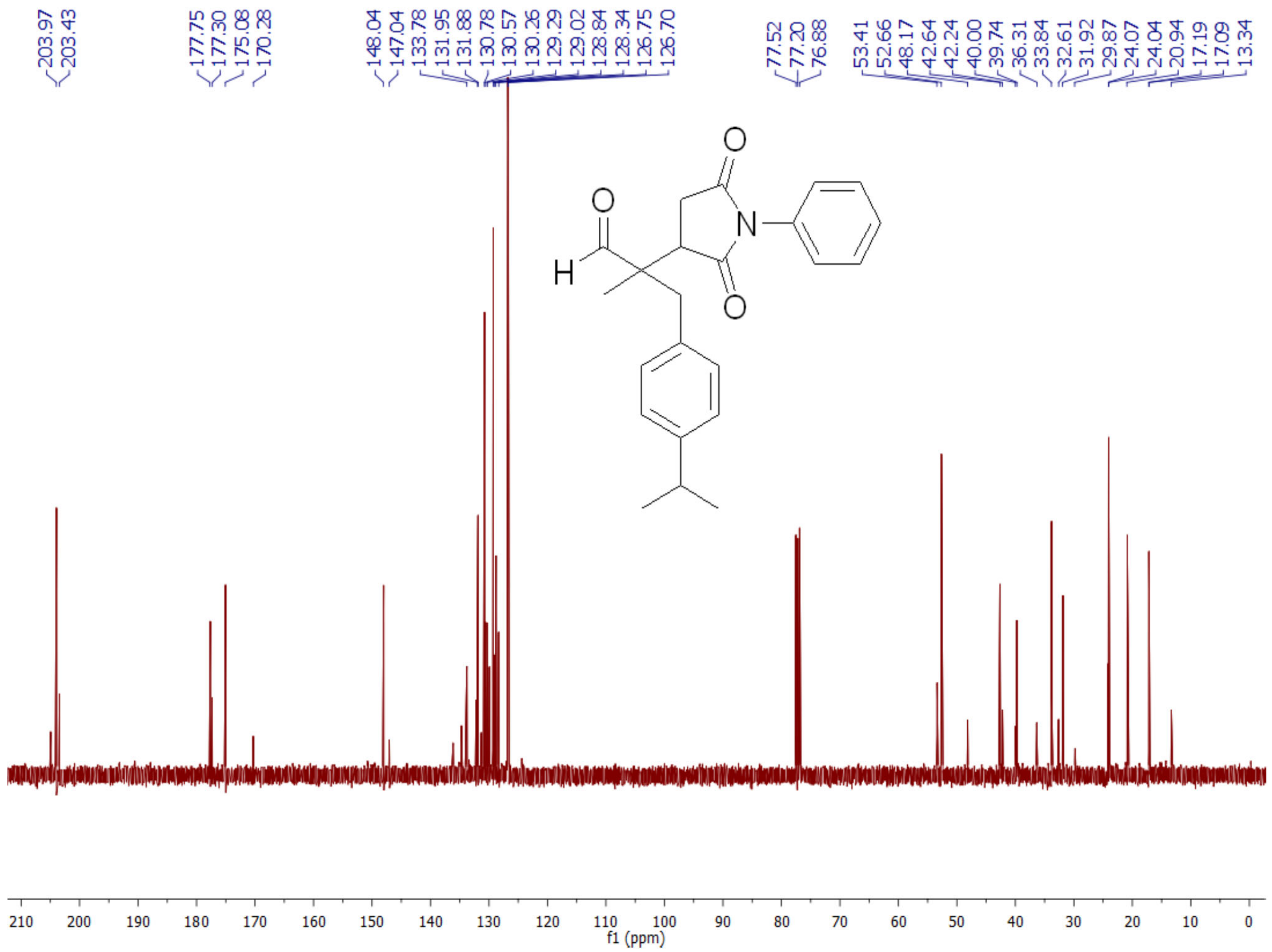

Figure S4. ^{13}C NMR spectrum of (2-(2,5-dioxo-1-phenylpyrrolidin-3-yl)-3-(4-isopropylphenyl)-2-methylpropanal) (**compound-1**).77.018

<Chromatogram>

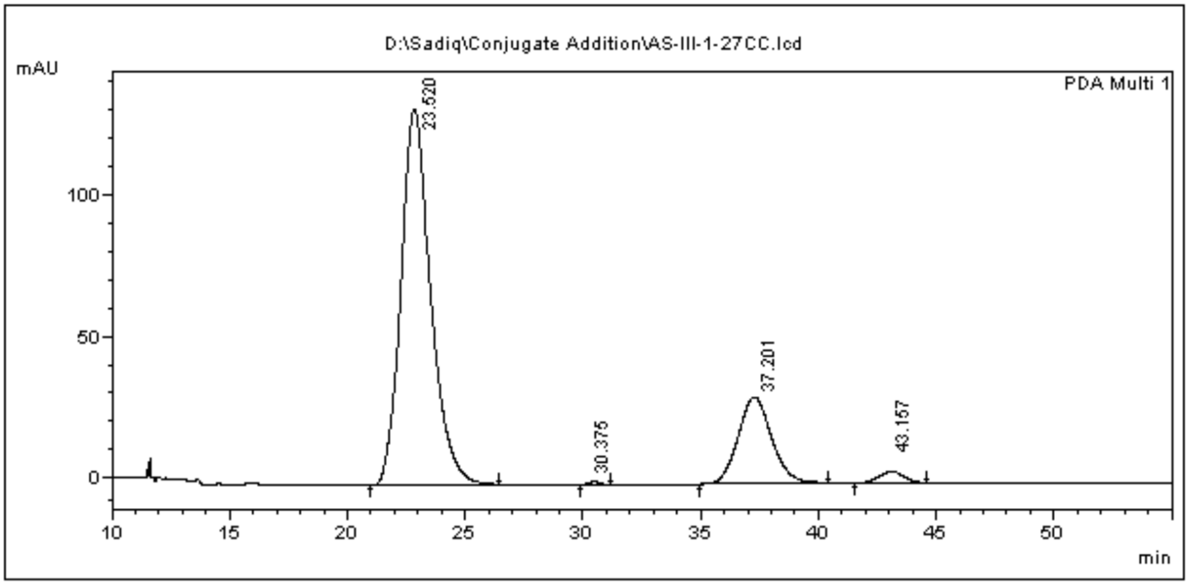

<Results>

PDA Ch1 210nm 4nm

Peak#	Ret. Time	Area	Area %	Mark
1	23.520	2992025	76.194	
2	30.375	32357	0.824	
3	37.201	706362	17.998	
4	43.157	195714	4.984	
Total		3926458	100.000	

Figure S5. Chiral HPLC chromatogram of (2-(2,5-dioxo-1-phenylpyrrolidin-3-yl)-3-(4-isopropylphenyl)-2-methylpropanal) (**compd-5**).

Biochemical assay

Troponin I (CTnI)

100 μl of standards, specimens, and controls were Dispensed into appropriate wells then 100 μl of Enzyme Conjugate Reagent was Dispensed into each well. Thoroughly mixed for 30 seconds and incubated at room temperature (18-25°C) for 90 minutes. The incubation mixture was removed by flicking plate contents into a waste container. The microtiter wells were rinsed and flicked 5 times with deionized water. All residual water droplets were removed by absorbent paper. 100 μl of TMB Reagent was Dispensed into each well, gently mixed for 5 seconds, and incubated at room temperature for 20 minutes. 100 μl of Stop Solution was to each well to stop the reaction, gently mixed for 30 seconds and the blue color changed to yellow color

completely. The absorbance was read at 450nm and mean absorbance values were found.

CK-MB (Creatine Kinase-Myocardial band)

The spectrophotometer was adjusted to 0 absorbances against distilled water. Working reagent 1ml was added to a cuvette (1 cm light path) then Sample serum 50 μL was added, mixed gently by inversion. Incubate for 5 minutes in the cell holder at 37 Ċ and record initial absorbance reading at 365 nm wavelength. Repeated the absorbance readings exactly after 1, 2, and 3 minutes, and the difference between absorbances was calculated. The mean value was found by ΔA/min, while ΔA represents the change in absorbance.

Creatine Kinase-Myocardial band activity in the sample was calculated by (U/L) = (◎A/min) x Factor, while the multiplying factor is 12000.

LDH (lactate dehydrogenase **)**

The spectrophotometer was adjusted to 0 absorbances against air. Working solution 1ml was added to a cuvette (1 cm light path) then Sample serum 20 μL was added, mixed gently by inversion. Incubate for 5 minutes in the cell holder at 37 Ċ and record initial absorbance reading at 340 nm wavelength. Repeated the absorbance readings exactly after 1, 2, and 3 minutes, and the difference between absorbance was calculated. The mean value was found by ΔA/min, while ΔA represents the change in absorbance.

Lactate dehydrogenase activity in the sample was calculated by **U/l = 8095 × ΔA at 340 nm/min**

AST/SGOT (Aspartate Aminotransferase)

The UV spectrophotometer was adjusted to 0 absorbances with distilled water. Working reagent 1ml was added to a cuvette (1 cm light path) then Sample serum 20 μL was added, mixed gently by inversion. . Incubate for 1 minute in cell holder at 37 Ċ and record initial absorbance reading at 340 nm wavelength. Repeated the absorbance

readings exactly after 1, 2, and 3 minutes, and the difference between absorbances was calculated. The mean value was found by ΔA/min, while ΔA represents the change in absorbance.

Aspartate Aminotransferase activity in sample was calculated by (U/L) = (ΔA/min) x Factor, while the multiplying factor is 1768.

ALT /SGPT (Alanine Transaminase)

The UV spectrophotometer was adjusted to 0 absorbances against reagent blank. Working reagent 1ml was added to a cuvette (1 cm light path) then Sample serum 20 μL was added, mixed gently by inversion. . Incubate for 5 minute in cell holder at 37 Ċ and record initial absorbance reading at 340 nm wavelength. Repeated the absorbance readings exactly after 1, 2, and 3 minutes, and the difference between absorbances was calculated. The mean value was found by ΔA/min, while ΔA represents the change in absorbance.

Alanine Transaminase activity in sample was calculated by (U/L) = (ΔA/min) x Factor, while the multiplying factor is 1746.

ALP (Alkaline Phosphatase)

The UV spectrophotometer was adjusted to 0 absorbances with distilled water. Working reagent 1ml was added to cuvette (1 cm light path) then Sample serum 20 μL was added, mixed gently by inversion. . Incubate for 1 minute in cell holder at 37 Ċ and record initial absorbance reading at 410 nm wavelength. Repeated the absorbance readings exactly after 1, 2, and 3 minutes, and the difference between absorbances was calculated. The mean value was found by ΔA/min, while ΔA represents the change in absorbance.

Alkaline Phosphatase activity in sample was calculated by (U/L) = (ΔA/min) x Factor while the multiplying factor at 37 Ċ is 2757.

TB (Bilirubin total)

The spectrophotometer was adjusted to 0 absorbances with distilled water. Working reagent 1ml was added to cuvette (1 cm light path) then Sample serum 100 µL was added, mixed gently by inversion. . Incubate for 10 minute in cell holder at 25 Ċ and recorded absorbance reading at 546 nm wavelengths. The concentration of TB was calculated as mg/dl by using formula.

mg/dL bilirubin in the sample= (A) Sample - (A) Sample Blank x 18

BD (direct Bilirubin)

The spectrophotometer was adjusted to 0 absorbances with distilled water. Working reagent 1ml was added to cuvette (1 cm light path) then Sample serum 100 µL was added, mixed gently by inversion. . Incubate for 5 minute in cell holder at 25 Ċ and record absorbance reading at 546 nm wavelengths for specimen against the blank standard. The concentration of DB was calculated as mg/dl by using formula.

mg/dL bilirubin = (A) Sample - (A) Sample Blank x 219

TC Total cholesterol (TC)

Working reagent 1ml was added to cuvette (1 cm light path) then Sample serum 10 µL was added, mixed gently by inversion. Incubate for 5 minute in cell holder at 37 Ċ and recorded the absorbance reading at 546 nm wavelengths for specimen against the blank standard. The concentration of TC was calculated as mg/dl by using formula.

$C=200 \times A_{(S)}/A_{(STD)}$ mg/dl

HDL-c (high density lipids)

Working reagent 1ml was added to a cuvette (1 cm light path) then Sample serum 100 µL was added, mixed gently by inversion. Incubate for 5 minutes in cell holder at 37 Ċ and recorded the absorbance reading at 546 nm wavelengths for specimen against the blank standard. The concentration of TC was calculated as mg/dl by using a formula.

HDL Cholesterol mg/dL = $A_{(TS)} / A_{(STD)}$ x25 x2 where 2 is dilution factor due to deprotenization

TG (Triglycerides)

The spectrophotometer wavelength was adjusted to 540 nm and the absorbance reading to zero with water as the reference. Triglyceride Working Reagent was warm to assay temperature. 1.0 ml of the Triglyceride Working Reagent was pipette into a cuvette. 10 µL (0.01 ml) of water, Glycerol Standard, and sample were added to cuvets labeled Blank, Standard, and Sample, respectively mixed by gentle inversion and Incubated for 5 minutes at 37 °C. Absorbance (A) of Blank, Standard, and Sample was recorded at 540 nm versus water as the reference. The absorbance of the Blank was subtracted from the absorbance of the Standard and the Sample to obtain a change in absorbance (A) due to triglycerides. Calculate the total triglyceride concentration of the sample by formula.

TG (Triglycerides) mg/dL = $A_{(TS)} / A_{(STD)}$ x concentration of standard

LDL-c (low density lipids)

Calculated by the **Friedewald equation**= **LDL-c = (Total Cholesterol) – (HDL-C) – (TGs/5)**

VLDL-c (Very low density lipids)

Calculated as the 20% of triglycerides
